# Identification of gene co-expression clusters in liver tissues from multiple porcine populations with high and low backfat androstenone phenotype

**DOI:** 10.1186/s12863-014-0158-8

**Published:** 2015-02-28

**Authors:** Sudeep Sahadevan, Ernst Tholen, Christine Große-Brinkhaus, Karl Schellander, Dawit Tesfaye, Martin Hofmann-Apitius, Mehmet Ulas Cinar, Asep Gunawan, Michael Hölker, Christiane Neuhoff

**Affiliations:** Institute of Animal Science, University of Bonn, Endenicher Alle, Bonn, 53115 Germany; Fraunhofer Institute for Algorithms and Scientific Computing (SCAI), Schloss Birlinghoven, Sankt Augustin, 53754 Germany; Department of Animal Science, Faculty of Agriculture, Erciyes University, Kayseri, Turkey; Department of Animal Production and Technology, Bogor Agricultural University, Bogor, Indonesia

**Keywords:** Boar taint, Androstenone, RNA-seq, Microarray, Multiple dataset, Co-expression, Cluster analysis, Androgen metabolism, Lipid metabolism

## Abstract

**Background:**

Boar taint is principally caused by accumulation of androstenone and skatole in adipose tissues. Studies have shown high heritability estimates for androstenone whereas skatole production is mainly dependent on nutritional factors. Androstenone is a lipophilic steroid mainly metabolized in liver. Majority of the studies on hepatic androstenone metabolism focus only on a single breed and very few studies account for population similarities/differences in gene expression patterns. In this work, we concentrated on population similarities in gene expression to identify the common genes involved in hepatic androstenone metabolism of multiple pig populations. Based on androstenone measurements, publicly available gene expression datasets from three porcine populations were compiled into either low or high androstenone dataset. Gene expression correlation coefficients from these datasets were converted to rank ratios and joint probabilities of these rank ratios were used to generate dataset specific co-expression clusters. Finally, these networks were clustered using a graph clustering technique.

**Results:**

Cluster analysis identified a number of statistically significant co-expression clusters in the dataset. Further enrichment analysis of these clusters showed that one of the clusters from low androstenone dataset was highly enriched for xenobiotic, drug, cholesterol and lipid metabolism and cytochrome P450 associated metabolism of drugs and xenobiotics. Literature references revealed that a number of genes in this cluster were involved in phase I and phase II metabolism. Physical and functional similarity assessment showed that the members of this cluster were dispersed across multiple clusters in high androstenone dataset, possibly indicating a weak co-expression of these genes in high androstenone dataset.

**Conclusions:**

Based on these results we hypothesize that majority of the genes in this cluster forms a signature co-expression cluster in low androstenone dataset in our experiment and that majority of the members of this cluster might be responsible for hepatic androstenone metabolism across all the three populations used in our study. We propose these results as a background work towards understanding breed similarities in hepatic androstenone metabolism. Additional large scale experiments using data from multiple porcine breeds are necessary to validate these findings.

**Electronic supplementary material:**

The online version of this article (doi:10.1186/s12863-014-0158-8) contains supplementary material, which is available to authorized users.

## Background

Boar taint is often described as an off odor or off taste noticeable from non castrated boar meat [1]. The accumulation of androstenone and skatole in porcine adipose tissues is one of the primary reasons for boar taint [2]. Studies have reported high heritability estimates of androstenone [3-5] whereas skatole synthesis is primarily dependent on nutritional factors and genetic control of skatole levels have not been reported [6]. Androstenone is a lipophilic sex pheromone synthesized in testis. One of the widely practiced methods of reducing boar taint is the surgical castration of boars, to limit the synthesis of androstenone [7]. European union has issued a declaration for the abolishment of piglet castration without anesthesia by 2018 on grounds of animal welfare [8]. One of the methods to reduce boar taint is selection and breeding of animals with reduced androstenone content in backfat. A prerequisite for developing breeding techniques and selecting genetic candidates to reduce boar taint is understanding the cellular mechanisms behind the synthesis and metabolism of androstenone. Androstenone is synthesized in testis and metabolized in liver [9]. Although testis is the site of androstenone synthesis in boars, this work focuses on the genetic factors involved in the metabolism of androstenone in liver. A number of researches have already tried to understand the cellular mechanisms behind the metabolism of androstenone in porcine liver [10-16]. In liver, metabolism of steroid hormones, xenobiotics and other endogenous compounds are mediated by phase I and phase II metabolic processes [17-20]. Studies on androstenone hepatic metabolism have come to the conclusion that phase I and phase II pathway enzymes are involved in the metabolism of androstenone in porcine liver and the majority of these studies were mainly focused on 3β-HSD, cytochrome P450 and sulfotransferase families of genes [6,9,11,13,15,21,22]. In this scenario, based on the information from the studies mentioned, two major points have to be taken into consideration: (i) except for a few candidate biomarkers, genetics behind metabolic pathways and enzymes involved in hepatic androstenone metabolism are largely unknown and (ii) most of the aforesaid studies except for [15] used only a single porcine breed to study the genetics behind androstenone metabolism. Studies have indicated that there are differences in the expression of genes from same tissue samples belonging to different breeds [15,23,24].

Since there are sizable gaps in our knowledge about the genetic mechanisms involved in hepatic androstenone metabolism, using a data driven approach incorporating gene expression data from a number of high throughput experiments in multiple populations on hepatic androstenone metabolism has a number of advantages: (i) by combining data from multiple populations it would be possible to understand the underlying population/breed similarities in genes governing androstenone metabolism, (ii) since the analysis includes data from multiple populations, the candidate biomarkers can be used to fill current gaps in the understanding of androstenone hepatic metabolism gene regulation and finally (iii) the analysis results could be used as a comparison standard to understand breed differences. This work is an attempt to explore the possibilities of combining metadata from multiple high throughput gene expression datasets to study the similarities in gene expression patterns and to identify the common genes involved in hepatic androstenone metabolism of three different porcine populations: a Duroc×*F*_2_ population and Duroc and Norwegian Landrace breeds. We limited our analysis to these three pig populations since it was not possible to obtain publicly available high throughput gene expression datasets on androstenone metabolism for any other pig breeds. The major aim of this work was to identify the similarities in gene expression patterns to determine the common genes involved in hepatic androstenone metabolism of three different pig populations using an integrative analysis approach and a state of the art clustering technique.

## Materials and methods

### Materials

#### Datasets

Three publicly available high throughput expression datasets were used in this work and all three expression datasets used in this experiment were generated to profile the gene expression differences between liver tissues of low and high androstenone (LA and HA) phenotypes (boars). Out of the three datasets used, one was from an in-house RNA-seq experiment performed on a sample commercial population of a Duroc sire line, Duroc×*F*_2_ boars [10]. In this experiment, liver samples from 5 boars with extreme high levels of androstenone measurement (2.48±0.56 μg/g) in backfat were categorized as high androstenone animals (HA) and liver samples from 5 boars with extreme low levels of androstenone measurement (0.24±0.06 μg/g) in backfat were categorized as low androstenone animals (LA). Additional details of library preparation, sample collection and sequencing are available in [10]. This dataset will be referred to as DuF2 dataset in further analysis steps. The remaining two datasets were from a microarray experiment based on a custom porcine cDNA microarray platform. In this experiment, gene expression profiling was performed on boar liver samples from two breeds, Duroc and Norwegian Landrace [15]. Expression profiling was performed separately for each breed and both datasets contained 29 HA animals and 29 LA animals each [15]. For HA Duroc animals the average androstenone level was 11.57±3.2 ppm and for LA Duroc animals, the average androstenone level was 0.37±0.17 ppm [15]. In case of Norwegian Landrace animals, average measurement of androstenone in HA animals was 5.95±2.04 ppm whereas the average androstenone level for LA animals was 0.14±0.04 ppm [15]. Further details of this experiment are available in [15]. The datasets from this microarray experiment will be referred to as Duroc and Landrace datasets in our analysis. The datasets were grouped into LA and HA datasets based on the classification of animals into low and high androstenone animals in the original experiments. Further details on animal selection and classification into high and low androstenone animals are available in the original experiments [10,15]. Table 1 gives additional details of the datasets used in our experiment.

**Table 1:** **Expression dataset details**

**Dataset**	**#Genes**	**#Common genes**	**#LA samples**	**#HA samples**	**Breed**	**GEO dataset id**	**GEO platform id**
DuF2	11,736	7,693	5	5	Duroc×*F*_2_	GSE44171	GPL11429
Duroc	11,186	7,693	29	29	Duroc	GSE11073	GPL6173
Landrace	11,186	7,693	29	29	Norwegian Landrace	GSE11073	GPL6173

Table giving details of expression dataset used in this work.

### Methods

#### Data set mapping, quality control and normalization

**RNA-seq data** The starting point of our analysis was the quality control mapping and normalization of DuF2 dataset. In the first quality control step, PCR primers and bad quality sequences (Phred score<20) reported by FASTQC quality control application [25] in RNA-seq raw read files (DuF2 dataset) were trimmed off. The raw reads after this filtration step were then mapped to the latest *Sus scrofa* genome build Sscrofa10.2 using the “splice aware” mapping algorithm TopHat [26]. In the final step, BEDTools [27] was used to compute the raw expression matrix (raw read count set) from the mapping files generated by the TopHat algorithm. A key difference between an expression matrix from an RNA-seq dataset and an expression matrix from microarray dataset is that the RNA-seq expression matrix follows a negative binomial distribution [28], whereas the expression matrix from microarray data follows a Gaussian distribution. Due to this difference in assumptions about the underlying data distributions, comparison/merging of expression results from these two different platforms are not straightforward. One of the recent advancements in the statistical analysis of RNA-seq data is an analysis method proposed by Law et al. [29]. This publication asserts that microarray like statistical methods can be applied to RNA-seq data after mean-variance modeling and log2 transformation [29]. The above mentioned data normalization method is implemented as “voom” function in limma R package [30]. Following the methodology proposed by Law et al. [29], we normalized and log2 transformed our RNA-seq expression matrix.

**Microarray data** The next step in our analysis was the retrieval, normalization and mapping of microarray expression data from Duroc and Landrace datasets to gene identifiers from Sscrofa10.2 gene build. The data normalization procedure described in the original microarray experiment is as follows: after hybridization and scanning, the mean foreground intensities were log transformed and normalized using print-tip loess normalization procedure in R [31] limma package [15]. Since the standard procedures of normalization were followed in the original experiment, we retrieved the normalized expression datasets from the corresponding GEO dataset using R package GEOQuery [32]. The distributions of DuF2 dataset before and after normalization and Duroc and Landrace datasets were visualized using density plots and these data distribution density plots are given in Additional file 1.

One of the challenges we faced in analyzing these microarray datasets (Duroc and Landrace datasets) together with our in-house RNA-seq dataset (DuF2 dataset) was the mapping between the custom probe ids used in the microarray platform and Entrez gene ids used in RNA-seq expression dataset. The cDNA microarray chip (see Table 1) used in the experiment was designed before the release of the pig genome [33] and used cDNA clones from Sino-Danish Pig Genome Sequencing Consortium as probes. Since these custom designed microarray probes and Entrez gene ids from RNA-seq dataset were not directly compatible, we generated a mapping between the microarray probe identifiers and NCBI Entrez gene identifiers. For this purpose, sequence alignments were performed between the FASTA sequences of these custom probes and Sscrofa10.2 Refseq cDNA sequences mapped to Entrez gene ids using NCBI standalone BLAST executable [34] (version: 2.2.28+, approach: all-vs-all and reciprocal blast). The Sscrofa10.2 sequence database generated for BLAST-ing consisted of 25,890 cDNA sequences mapped to Entrez gene ids and the microarray probe sequence database was comprised of 26,877 sequences. In this step, we generated mapping between 11,251 microarray cDNA probes and 11,186 Entrez gene ids. In order to avoid the conflicts where multiple cDNA probes were mapped to an Entrez gene id, the expression values from the probe with the largest variance between sample expression values was mapped to the corresponding Entrez gene id and the remaining conflicting probe ids and expression values were discarded from further analysis.

At the end of mapping and normalization of DuF2, Duroc and Landrace datasets only 7,693 genes were common between all these datasets. Hence, the expression values from only these genes were retained in all the datasets for further analysis. In the next step, we regrouped the expression matrices according the phenotype assignment and generated 2 expression matrix sets: an LA set and an HA set with 3 expression matrices each. A schematic representation of the entire workflow used in this analysis is given in Additional file 2.

#### Generating multi population co-expression networks

In this study, Pearson correlation coefficient between gene pairs in an expression matrix was used as a measure of co-expression. The principal aim behind this experiment was to generate signature gene co-expression networks by merging metadata from multiple gene expression datasets to study porcine hepatic androstenone metabolism. Stuart et al. [35], developed a method for computing gene co-expression clusters across microarray datasets from multiple species. In this method, the authors calculated correlation coefficient between gene pairs in each dataset and further computed rank order statistics for each gene pair [35]. The rank order statistics for each gene pair (each unique correlation coefficient) was calculated as the ratio of its rank in ordered correlation coefficients to the total number of gene pairs (unique correlation coefficients). Finally, the joint cumulative density function (joint cdf) of an n-dimensional rank order statistics was calculated using the equation:

$$P(r_{1},r_{2},\dotsi,r_{n})=n! \int_{0}^{r_{1}} \int_{r_{1}}^{r_{2}} \dotsi \int_{s_{n-1}}^{r_{n}}d_{s1},d_{s2},\dotsi,d_{sn} $$

[35].

In this equation *n* is the number of species in the study and *r*_1_,*r*_2_, ⋯,*r*_*n*_ are the rank order ratios of a gene pair in multiple species (datasets). In this work, we adopted the aforesaid approach proposed by Stuart et al. [35] to generate the signature co-expression networks related to porcine hepatic androstenone metabolism. As a first step for this purpose, Pearson correlation coefficients were calculated for gene pairs in all the 6 expression matrices (3 LA and 3 HA expression matrices) separately. Since we had 7,693 (n = 7,693) common genes among all our datasets, we ended up with 29.5 million unique gene pairs $\mathrm {\left (\frac {n\times (n-1)}{2}\right)}$ per dataset. Based on the initial experiments (data not shown) we discovered that due to this high number of unique correlation coefficients, using signed values of correlation coefficients for rank order calculation would result in high rank order ratios even for correlation coefficients with a very small positive value. Since these rank ratios are used for computing the joint cdf, even the gene pairs with very small positive correlation coefficients in all the three expression matrices of a dataset would receive a high joint cumulative probability. Since our aim was to generate holistic co-expression networks for LA and HA phenotypes, we used the absolute value of correlation coefficients to compute the rank order statistics of gene pairs. After calculating the rank order ratios of gene pairs in all the expression matrices, gene pair correlation coefficients and rank order ratios were compiled into either LA or HA set according to the phenotype assignment described in the previous subsection.

In the next step, we trimmed off gene pairs with correlation coefficients ≤+0.50 in LA and HA sets separately. This pruning step was aimed at removing all those gene pairs with conflicting directionalities (positive correlation in one or two datasets and negative correlation in the other) and very small positive correlation coefficients. This step was performed to ensure that in the final step, the correlation coefficients between all the gene pairs in a cluster are positive and high in LA and HA clusters. After this pruning process, the number of remaining gene pairs in LA and HA sets were 43,480 (from 3,648 genes) and 42,309 (from 2,826 genes) respectively. The joint cumulative probability of rank order ratios for these gene pairs in LA and HA sets were calculated using the equation stated above. Using these cumulative probabilities as edge weights for LA and HA gene pairs we generated two phenotype specific edge weighted co-expression networks: an LA network with 43,480 edges among 3,648 nodes and an HA network with 42,309 edges and 2,826 nodes. These LA and HA co-expression networks were further used as inputs for graph clustering and community detection. These steps are described in detail in the next subsection.

#### Identifying statistically significant co-expression clusters

For identifying the gene clusters in LA and HA co-expression networks, we used a graph clustering algorithm known as Infomap [36]. Infomap clustering algorithm is based on an information theoretic method called map equation. This clustering algorithm is based on optimizing the problem of compressing the information within a network structure and finding regular patterns in a network structure that generate the information [36]. A benchmark test [37] conducted on multiple graph clustering and community detection algorithms concluded that Infomap algorithm has a reliable performance in a number of real world scenarios. Based on this conclusion in [37], we chose Infomap clustering algorithm for clustering LA and HA co-expression networks.

Although Infomap was shown to be one of the best performing clustering algorithms, the clustering outputs from the algorithm is still not deterministic. Like a number of other graph clustering algorithms [38-41], even if all the parameters supplied to the algorithm are kept constant, clustering solutions can still vary slightly depending on the random seed (random number) chosen to initiate clustering. A solution to this problem is a clustering strategy known as consensus clustering [42-45]. The basic principle behind consensus clustering is identifying the general agreement (consensus) between a number of different clustering solutions. Recently, Lancichinetti and Fortunato [42] proposed a greedy algorithm for consensus clustering. This algorithm generates a matrix (consensus matrix) based on the co-occurrence of nodes in clusters belonging to a number different of input clustering solutions (from the same clustering algorithm) and uses this consensus matrix as an input for the original clustering method, thus leading to a new set of clusters. This process is iterated until a complete consensus solution is reached, which upon further clustering would not result in additional clusters [42].

**Figure 1:**
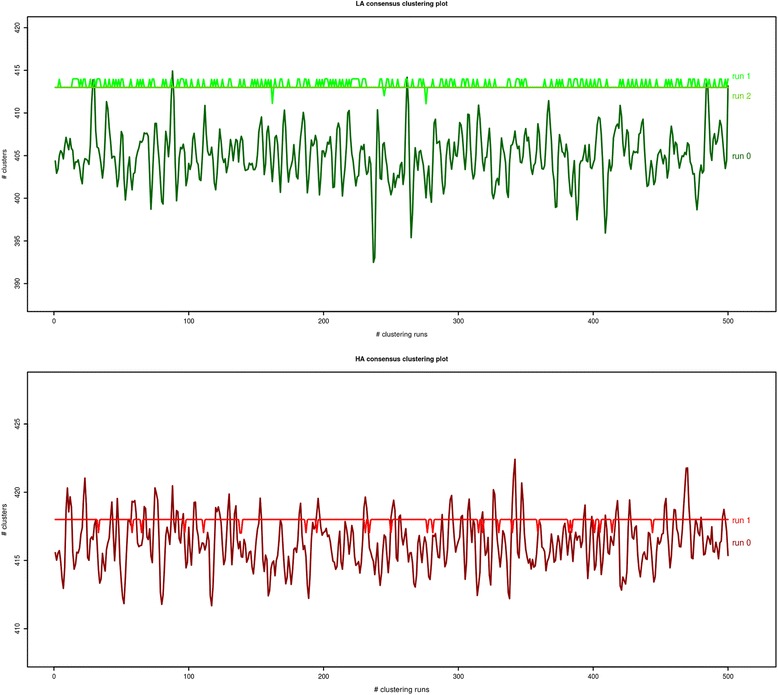
**LA HA networks consensus clustering.** Legend: “run 0” in both graphs indicate first clustering run using LA and HA networks, “run 1” indicates clustering run for the first consensus cluster and “run 2” indicates clustering run for the second consensus cluster.

In our work, a combination of Infomap clustering algorithm and consensus clustering technique was used to cluster LA and HA co-expression networks. All the input parameters, except the random seed were kept constant for clustering LA and HA networks and 500 clustering solutions were generated in each iteration (per network). Complete consensus clusters were generated from LA network after 3 iterations whereas complete consensus clusters were generated from HA network after only 2 iterations. Figure 1 gives an overview of the LA and HA consensus clustering runs and the total number of clusters generated per run for each network.

Although consensus clustering technique can enhance the accuracy and reliability of the resulting clusters, this method still cannot guarantee the significance of a cluster with respect to the input network. Since our initial LA and HA co-expression networks had a large number of nodes (3,648 and 2,826 respectively), it could be possible that some of the clusters generated from these networks are not specific to the phenotype at all, but random collections of nodes either as a result of the large number of nodes in the initial networks or as a result of an artifact in the cluster algorithm. In this work, we intended to select only the clusters which were not random but specific to the given input network. So, in the next step, we performed a cluster clean up process and assessment of the statistical significance of the clusters by applying the methodology proposed by [38]. This methodology is based on the assumption that given a graph (network) and clusters generated from the graph, the statistical significance of clusters can be estimated as the probability of finding these clusters in random null model graphs generated from the original graph and that a statistical significance cut-off can be used to identify non random clusters. The authors also proposed a cluster clean up procedure, where the nodes are ranked according to the probability of inclusion in a cluster (when compared to a null model) and only the nodes with probability above a certain significance threshold are kept in the pruned cluster [38]. We adopted this methodology to perform cluster clean up and statistical significance estimation of LA and HA co-expression networks. After this step, clusters with less than 10 nodes and significance score (p-value) ≥0.05 were excluded from further analysis.

#### Enrichment analysis

To identify and describe the biological functions of these significant co-expression networks we performed Gene Ontology (GO) and KEGG enrichment analysis for each cluster. Since we were only interested in the biological functions of these clusters, GO enrichment analysis was limited to the biological process sub tree of the Gene Ontology. GO enrichment analysis was performed using the R package topGO [46]. The algorithm used by topGO package takes into account the hierarchical structure of GO graph and shares annotations between parent and child nodes of the graph for significance testing using Fisher’s exact test [47]. KEGG enrichment analysis was performed using a custom R script and Fisher’s exact test was used for testing the significance of KEGG annotated pathways. In both of these enrichment analyses, only the GO terms/KEGG pathways with significance p-value <0.05 and with ≥ 5 annotated genes were selected as significantly enriched.

#### Cluster similarity analysis

Once we identified the significant clusters in our networks and performed enrichment analysis, the next step was to calculate the similarity between these significant LA and HA clusters. In this step, we calculated the physical and functional similarity between significant LA and HA clusters. It should be noted that the physical similarity was calculated for all significant LA and HA clusters whereas functional similarity was calculated only for the clusters with GO enrichment.

**Physical similarity** Physical similarity between LA and HA clusters were calculated using a hypergeometric test. For each significant LA cluster, an HA cluster was retrieved and hypergeometric test was performed between the nodes of these clusters to identify the overlap. In this step, only LA - HA similarity was tested since Infomap clustering algorithm generates non overlapping clusters. P-values were generated using the phyper function in R environment and the hypergeometric test results were pruned at a significance threshold of p-value<0.05.

**Functional similarity** Functional similarity between LA and HA significant clusters was established by calculating the Gene Ontology semantic similarity [48-50]. In this step, we were interested only in assessing the functional similarity between those clusters showing significant GO enrichment in the enrichment analysis step. For a given set of genes, GO semantic similarity can be calculated based on the number of shared Gene Ontology annotations between the genes. Gene ontology based semantic similarity can be assessed by two main methods, (i) Information content based methods [49,51-53] and (ii) Graph based methods [50].

In this work, GO semantic similarity was calculated between the significantly enriched GO terms of all the clusters obtained from the enrichment analysis step. We refer to the GO semantic similarity obtained in this step as functional similarity between two clusters, since the semantic similarity calculated directly reflects the relationship between enriched GO biological process terms of two clusters and hence is a measurement of the biological functional relationship. For calculating the semantic similarity between GO terms, we used the graph based Wang method [50] as implemented in GOSemSim [54] bioconductor package. In this step, semantic similarity was calculated between all enriched LA and HA clusters. For enriched GO terms in each LA or HA cluster, GO terms from another LA or HA cluster was drawn and semantic similarity was calculated between these terms using Wang method and these similarity measurements were combined into a single value using best-match average strategy (BMA) [54]. These semantic similarity values were termed *sim*_*CLUS*_ for future references.

Although the step mentioned above allows to calculate semantic similarity between two enriched clusters in our analysis, this step does not provide a cut-off threshold to indicate whether the similarity between the two clusters were significant or not. To provide a significant cut-off point for semantic similarity, we followed an empirical approach based on random sampling. In this step, we retrieved all GO biological process annotations for porcine genes and randomly sampled two sets of GO terms from these annotations. The number of sampled terms was also kept random and was drawn from the number of GO terms enriched for either LA or HA clusters. GOSemSim package was again used to calculate semantic similarity. This whole step was repeated 10,000 times to generate a set of random semantic similarity measures. These random semantic similarity values were termed as *sim*_*RAND*_ for further references. Finally, the significance threshold cut-off empirical p-value for each *sim*_*CLUS*_ was calculated as:

$${} {Pval}_{Empricial}=\frac{\#\ {sim}_{RAND}>{sim}_{CLUS}}{N}, \mathrm{where\,N=10,000.} $$

The threshold cut off used here was *Pval*_*Empricial*_<0.05. In the next step, we generated two cluster similarity graphs based on physical similarity assessment and functional similarity assessment. These graphs were visualized using the biological network visualizing platform, Cytoscape [55].

## Results and discussion

In our analysis, a total of 17 clusters from LA co-expression network and 12 clusters from HA co-expression network were found be significant with more than 10 nodes per cluster. Table 2 shows the number of genes, significance score and average correlation coefficients of nodes in these clusters across three datasets. A comparison of correlation coefficients in the three datasets shows that the correlation coefficient values were comparatively higher in Duroc×*F*_2_ (RNA-seq) dataset (Table 2). The maximum and minimum number of nodes (genes) in LA co-expression clusters were 478 and 20 respectively whereas the maximum and minimum number of nodes in HA co-expression clusters were 616 and 11 respectively (Table 2). In case of DuF2 dataset, we think that the higher correlation coefficient is mainly the combined result of sensitivity of the RNA-seq technique and the normalization procedure. RNA-seq being a more sensitive technique might have given a high expression value per gene. Since all the expression values (read count) were large positive numbers, the log2 transformation also tend to give largely positive values which could have impacted the correlation coefficient calculations. Seven LA co-expression clusters and 5 HA co-expression clusters were enriched for GO biological processes terms, whereas 5 LA co-expression clusters and 3 HA co-expression clusters were enriched for KEGG metabolic pathways. Table 3 gives an overview on the number of GO terms and KEGG pathways enriched per cluster. The results from GO and KEGG enrichment analysis show that LA and HA co-expression clusters are involved in a number of divergent biological functions. Further details of GO and KEGG enrichment analysis, such as enriched terms, number of enriched genes, p-value of enrichment and gene ids of enriched genes are given in Additional files 3 and 4.

**Table 2:** **Significant clusters in LA and HA co-expression networks**

**Cluster Id**	**#Genes**	**Significance (p-value)**	**DuF2 cor. coeff. (mean ± sd)**	**Duroc cor. coeff. (mean ± sd)**	**Landrace cor. coeff. (mean ± sd)**
LA 0	478	0.00216	0.758 ± 0.138	0.850 ± 0.115	0.625 ± 0.090
LA 1	316	0.00267	0.742 ± 0.135	0.832 ± 0.122	0.622 ± 0.091
LA 2	134	0.0076	0.776 ± 0.139	0.672 ± 0.100	0.596 ± 0.075
LA 3	116	0.02248	0.741 ± 0.133	0.849 ± 0.111	0.630 ± 0.089
LA 4	96	0.04911	0.773 ± 0.139	0.666 ± 0.101	0.600 ± 0.074
LA 6	86	0.01046	0.793 ± 0.149	0.714 ± 0.108	0.600 ± 0.070
LA 7	87	0.0203	0.736 ± 0.143	0.724 ± 0.115	0.582 ± 0.063
LA 8	72	0.0379	0.765 ± 0.134	0.707 ± 0.132	0.587 ± 0.069
LA 9	68	0.01526	0.765 ± 0.149	0.610 ± 0.081	0.605 ± 0.084
LA 11	61	0.01415	0.729 ± 0.141	0.663 ± 0.126	0.662 ± 0.096
LA 12	40	0.04167	0.739 ± 0.125	0.622 ± 0.085	0.598 ± 0.074
LA 14	39	0.00594	0.736 ± 0.139	0.700 ± 0.116	0.610 ± 0.076
LA 15	30	0.04776	0.768 ± 0.138	0.641 ± 0.104	0.592 ± 0.065
LA 17	21	0.01309	0.748 ± 0.139	0.676 ± 0.131	0.612 ± 0.077
LA 18	28	0.00258	0.749 ± 0.134	0.661 ± 0.117	0.591 ± 0.075
LA 19	20	0.00408	0.726 ± 0.122	0.679 ± 0.100	0.622 ± 0.080
LA 21	21	0.01807	0.758 ± 0.140	0.746 ± 0.107	0.620 ± 0.084
HA 0	616	0.03963	0.780 ± 0.139	0.704 ± 0.115	0.663 ± 0.102
HA 1	75	0.0166	0.812 ± 0.132	0.598 ± 0.077	0.668 ± 0.106
HA 3	23	0.0023	0.815 ± 0.128	0.612 ± 0.081	0.679 ± 0.109
HA 4	18	0.00095	0.826 ± 0.117	0.597 ± 0.065	0.622 ± 0.079
HA 10	207	0.00203	0.770 ± 0.137	0.741 ± 0.116	0.681 ± 0.114
HA 11	22	0.01025	0.773 ± 0.125	0.775 ± 0.098	0.656 ± 0.103
HA 12	13	0.01196	0.776 ± 0.138	0.747 ± 0.105	0.660 ± 0.090
HA 14	75	0.00429	0.750 ± 0.141	0.611 ± 0.086	0.685 ± 0.100
HA 17	40	0.01279	0.821 ± 0.133	0.637 ± 0.088	0.619 ± 0.085
HA 18	25	0.02743	0.770 ± 0.136	0.776 ± 0.094	0.735 ± 0.101
HA 19	25	0.02149	0.767 ± 0.128	0.604 ± 0.080	0.680 ± 0.106
HA 22	11	0.04384	0.744 ± 0.136	0.677 ± 0.121	0.689 ± 0.105

This table contains information on significant clusters generated from LA and HA co-expression networks.

**Table 3:** **Enrichment statistics of significant LA and HA coexpression clusters**

**Cluster Id**	**#GO enriched terms**	**#KEGG enriched pathways**
LA 0	19	–
LA 1	10	–
LA 2	14	11
LA 3	5	3
LA 4	–	1
LA 6	8	1
LA 7	4	–
LA 8	5	–
LA 9	–	2
HA 0	50	5
HA 1	7	6
HA 3	3	–
HA 10	8	–
HA 17	3	2

This table contains information on the number of GO terms and KEGG pathways enriched in significant clusters generated from LA and HA co-expression networks.

Although several LA and HA clusters were enriched for GO processes and KEGG pathways, based on enrichment results, we selected LA cluster 2 for a detailed analysis. LA cluster 2 GO and KEGG enrichments are complimentary to each other and strongly points to the involvement of the member genes in phase I and II metabolism and the metabolism of steroid hormones and drugs. This cluster was enriched for GO processes such as oxidation-reduction process, xenobiotic metabolic process, triglyceride metabolic process, lipid metabolic process, cholesterol metabolic process, response to drug, response to hormone stimulus (Table 4) as well as KEGG pathways such as PPAR signaling pathway, peroxisome, retinol metabolism, drug metabolism - other enzymes, drug metabolism - cytochrome P450 and metabolism of xenobiotics by cytochrome P450 (Table 5). Additional information on GO and KEGG enrichments are available in Additional files 3 and 4. It was previously established that steroid metabolism is closely linked to metabolism of drugs/xenobiotics and that the metabolism of steroids, steroid hormones, drugs and other xenobiotics are mediated by phase I and phase II metabolic pathways [17-20]. One of the GO biological processes enriched in LA cluster 2 results is the oxidation reduction process and it was already found that oxidation and reduction metabolic processes constitute to phase I metabolism [56]. Several genes involved in xenobiotic metabolism are also involved in the metabolism of androgens [57] and GO biological process “xenobiotic metabolic processes” was enriched for LA cluster 2 (Table 4). In GO and KEGG enrichment results GO term aromatic compound catabolic process and KEGG pathways drug metabolism - cytochrome P450 and metabolism of xenobiotics by cytochrome P450 were enriched (Tables 4 and 5). Cytochrome P450 related enzyme pathways were identified to be involved in metabolism of aromatic compounds, drugs and steroid hormones [58,59].

**Table 4:** **LA cluster 2 GO enrichment**

**GO.ID**	**Term**	**#Enriched genes**	**Enrichment p-value**
GO:0055114	Oxidation-reduction process	42	9.6E-011
GO:0051289	Protein homotetramerization	6	0.0000016
GO:0006805	Xenobiotic metabolic process	8	0.000012
GO:0006641	Triglyceride metabolic process	5	0.002
GO:0006629	Lipid metabolic process	33	0.00231
GO:0009058	Biosynthetic process	40	0.01118
GO:0048869	Cellular developmental process	11	0.0115
GO:0006810	Transport	34	0.01378
GO:0008203	Cholesterol metabolic process	7	0.01502
GO:0042493	Response to drug	8	0.01503
GO:0046395	Carboxylic acid catabolic process	11	0.02834
GO:0019439	Aromatic compound catabolic process	14	0.02987
GO:0006869	Lipid transport	5	0.03686
GO:0009725	Response to hormone stimulus	7	0.04158

This table contains enriched GO biological process terms for LA cluster 2 genes.

**Table 5:** **LA cluster 2 KEGG enrichment**

**KEGG.ID**	**Pathway**	**#Enriched genes**	**Enrichment p-value**
ssc00982	Drug metabolism - cytochrome P450	9	0.00000325
ssc00071	Fatty acid degradation	8	0.00001695
ssc00980	Metabolism of xenobiotics by cytochrome P450	7	0.00019518
ssc00830	Retinol metabolism	7	0.00026192
ssc00053	Ascorbate and aldarate metabolism	5	0.00033240
ssc05204	Chemical carcinogenesis	7	0.00082319
ssc00983	Drug metabolism - other enzymes	5	0.00107901
ssc04146	Peroxisome	8	0.00109469
ssc00280	Valine, leucine and isoleucine degradation	6	0.00149421
ssc00380	Tryptophan metabolism	5	0.00343914
ssc03320	PPAR signaling pathway	6	0.00990966

This table contains enriched KEGG pathways for LA cluster 2 genes.

### LA cluster 2 gene functions

**Figure 2:**
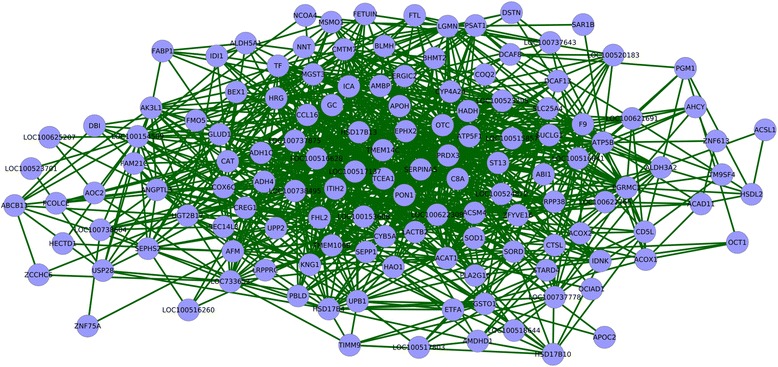
**LA cluster 2.** Figure showing the genes co-expressed in LA cluster 2. Legend: light blue nodes indicate genes and green edges indicate node co-expression (cor≥+0.50 in all three porcine populations).

LA cluster 2 was comprised of 134 nodes (genes) and 1,121 edges (Figure 2). Additional file 5 contains Cytoscape.xgmml network representation of this cluster and each edge in this cluster is annotated with correlation coefficients from all the three datasets and joint cumulative density probability calculated. Node degree calculations done on the cluster indicated that genes such as PRDX3, LOC100622308 (SCP2), LOC100516628 (UGT2B18-like), PON1 and OTC were the top ranking highly connected nodes in the cluster. Some of the major families of genes in this cluster were: the UGT gene family (UGT2B17, LOC100516628 (UGT2B18-like), LOC100738495 (UGT2B31-like), HSD/SDR gene family (HSD17B4, HSD17B10, HSD17B13, HSDL2), SLC gene family (LOC100737875 (SLC22A10), SLC25A4), ALDH gene family (ALDH3A2, ALDH5A1) and USP gene family (Usp9x, USP28) (see Figure 2). Since describing the functions of all the genes in LA cluster 2 would be beyond the scope of this manuscript, the gene discussion part is limited to a handful important genes described below.

Literature references show that UGT, HSD and ALDH gene families are associated with steroids and steroid hormone metabolism [60-62]. Three members of the UGT gene family, UGT2B17, LOC100516628 (UGT2B18-like) and LOC100738495 (UGT2B31-like) were co-expressed in LA cluster 2. Members of the UGT gene family are involved in the metabolism of steroids, biogenic amines, fat soluble vitamins, drugs and xenobiotics [63-65]. UGT2B17 was found to be important for hepatic detoxification and involved in androgen metabolism [66,67]. It was shown that UGT2B18 was predominantly active on C19 steroids with a hydroxyl group at the 3α position [68]. Kojima and Degawa demonstrated that UGT2B31 expression was higher in male pigs when compared to female pigs and that testosterone treatment of castrated boars increased UGT2B31 expression [69]. Canine UGT2B31 catalyzed the glucuronidation of compounds such as steriods, opoids, apliphatic alcohols and phenols [70]. Glucoronic acid, the substrate molecule for UGT glucuronidation process is a carboxylic acid. Since GO carboxylic acid catabolic process was enriched in LA cluster 2 results along with other metabolic processes such as xenobiotic metabolic process and cholesterol metabolic process (Table 4), it could be assumed that carboxylic acid (glucoronic acid) catabolism is interlinked with the metabolism of steroids, drugs and xenobiotics in the glucuronidation process. Considering that the literatures cited above points to steroid metabolic roles of these genes and that these genes were co-expressed in all the three LA datasets, it could be possible that the UGT family genes mentioned above were involved in androgen/androstenone metabolism in all the three datasets (population). In addition to UGT gene family, 4 members of HSD gene family were also co-expressed in our results. These genes are: HSD17B4, HSD17B10, HSD17B13 and HSDL2. Among these genes, three (HSD17B4, HSD17B10, HSD17B13) are members of 17β-HSD gene family. The reduction reactions catalyzed by 17β-HSDs are necessary for the formation of active androgens whereas the oxidative reactions inactivates potent sex steriods [71]. The enzyme encoded by gene HSD17B4 functions as a steroid inactivating enzyme and is also involved in the beta oxidation of fatty acids [72]. Additionally, it was also demonstrated that the conversion of Δ 5-androstene-3-17-diol to dehydro-epiandrosterone (DHEA) was inactivated by HSD17B4 [73]. HSD17B10 was shown to be expressed in human liver, gonads, localized to mitochondria and associated with phase I metabolic pathway. The mitochondrial ability to modulate intracellular levels of active sex steroids stem from this localization of HSD17B10 [74]. HSD17B13 is expressed in liver across a number of mammalian species. While the functions of HSD17B4 and HSD17B10 could be discussed in detail, we were unable to find published evidences related to HDS17B13. But, in the light of evidences from SDR (HSD) gene family, it could be hypothesized that HSD17B13 is also involved in the metabolism of sex steroids. Another short chain reductase (SDR/HSD) family member HSDL2 was found to be involved in cholesterol metabolism and homeostasis [75]. In case of SLC family genes in LA cluster 2, we found that LOC100737875 (SLC22A10) gene product transports sulfate conjugates of steroids, estrone sulfate and dehydroepiandrosterone sulfate (DHEAS) with high affinity [76]. We were unable to find any function for SLC25A4 with regard to androgen or sterid metabolism or transport. In case of ALDH gene family, although ALDH3A2 is involved in phase I metabolic pathway, known to catalyze the oxidation of long-chain aliphatic aldehydes to fatty acid and ALDH5A1 is involved in γ aminobutyric degradation [77], we could not find any evidences to link these genes to hepatic androgen/androstenone metabolism.

Another LA cluster 2 member, AKR1C1 is an NADPH dependent ketosteroid reductase. The product of this gene converts progesterone to its inactive form 20−*α*−*dihydroxyprogesterone* [78]. In androgen metabolism, the conversion of dihydrotestosterone (DHT) to 5α-androstane-3β,17β-diol is mainly catalyzed by AKR1C1 gene product [79]. It was also shown that AKR1C1 activity can be induced by phase II enzyme inducers [80], suggesting a potential role of this gene in phase II metabolic processes. FMO5 was another co-expressed gene in LA cluster 2. The enzyme encoded by this gene is NADPH dependent, upregulated by progesterone and catalyzes the oxidation of drugs, pesticides and xenobiotics [81]. It was also found that FMO5 is expressed in human liver cells and ≥50% of all FMO transcripts in human liver cells are from FMO5 [82]. STARD4, an LA cluster 2 member is widely expressed in liver and is demonstrated to be an important effector of lipid distribution in body [83]. Rodriguez-Agudo et al. [84] postulated that STARD4 might reduce steroid hormone production during murine development and another study [85] found that STARD4 functions in a rate limiting step in cholesterol ester formation. According to [86] STARD4 increases intracellular cholesteryl ester formation and is a major component of cholesterol homeostasis regulating mechanism. In our results, the gene ADH1C was also found to be co-expressed in LA cluster 2. This gene is a member of the alcohol dehyrogenase family which metabolize substrates such as ethanol, retinol, hydroxysteroids and lipid peroxidation products. A study done on human ADH1C allele 2 found that this allele (ADH1C*2) had measurable activity on steroidogenic compounds such as 5β-androstan-17β-ol-3-one, 5β-androstan-3β-ol-17-one, 5β-pregnan-3β-ol-20-one and 5β-pregnan-3,20-dione [87].

PGRMC1, a progesterone steroid receptor is an LA cluster 2 member predominantly expressed in liver and kidney. This gene was found to be involved in sterol metabolism/homeostasis and cell survival [88]. DBI, another LA cluster 2 member gene boost steroid synthesis by stimulating delivery of cholesterol to inner mitochondrial membranes [89]. The functional roles of DBI include supporting energy metabolism, transcription, membrane production and steroidogenesis [90]. According to [91], CRYZ gene, another LA cluster 2 member is associated with lipid, fatty acid and steroid metabolism. LOC100622308 (SCP2) gene encodes sterol carrying protein 2 and is also an LA cluster 2 member. This gene is found to be involved in hepatic cholesterol metabolism, biliary lipid secretion, and intracellular cholesterol distribution [92] and it is suggested that SCP2 might be involved in regulating steroidogenesis [93]. Yet another LA cluster 2 member gene in our analysis was LOC100523701 (aldehyde oxidase like). The richest source of this gene product in terms of transcriptome abundance is liver and is found in a number of mammals. Moreover, aldehyde oxidases are involved in phase I metabolism of a number of compounds and probably functions along with the microsomal cytochrome P450 system [94]. FHL2, another LA cluster 2 co-expressed gene is an androgen responsive gene and a co-activator of androgen receptor (AR) [95,96]. Further research also found that FHL2 is involved in steroid hormone related pathways and interacts with endoplasmic reticulum (ER) in the presence of 17β-estradiol [97]. An LA cluster 2 member gene, OCT1 interacts with AR and can interact with HNF1 to modulate its capacity to upregulate UGT2B expression in liver [57]. Since three UGT2B genes (UGT2B17, LOC100516628 (UGT2B18-like), LOC100738495 (UGT2B31-like)) and OCT1 are found in the same cluster and co-expressed in three different datasets (population), the potential action of OCT1 on UGT2B genes and their role in androgen/androstenone metabolism could be further investigated. Another LA cluster 2 coexpressed gene was PON1. PON1 is synthesized in liver and is involved in the biotransformation of various xenobiotics as well as protection against lipid peroxidation [98]. The next part of this section describes and discusses the results from cluster similarity assessments.

### Cluster similarity analysis

**Figure 3:**
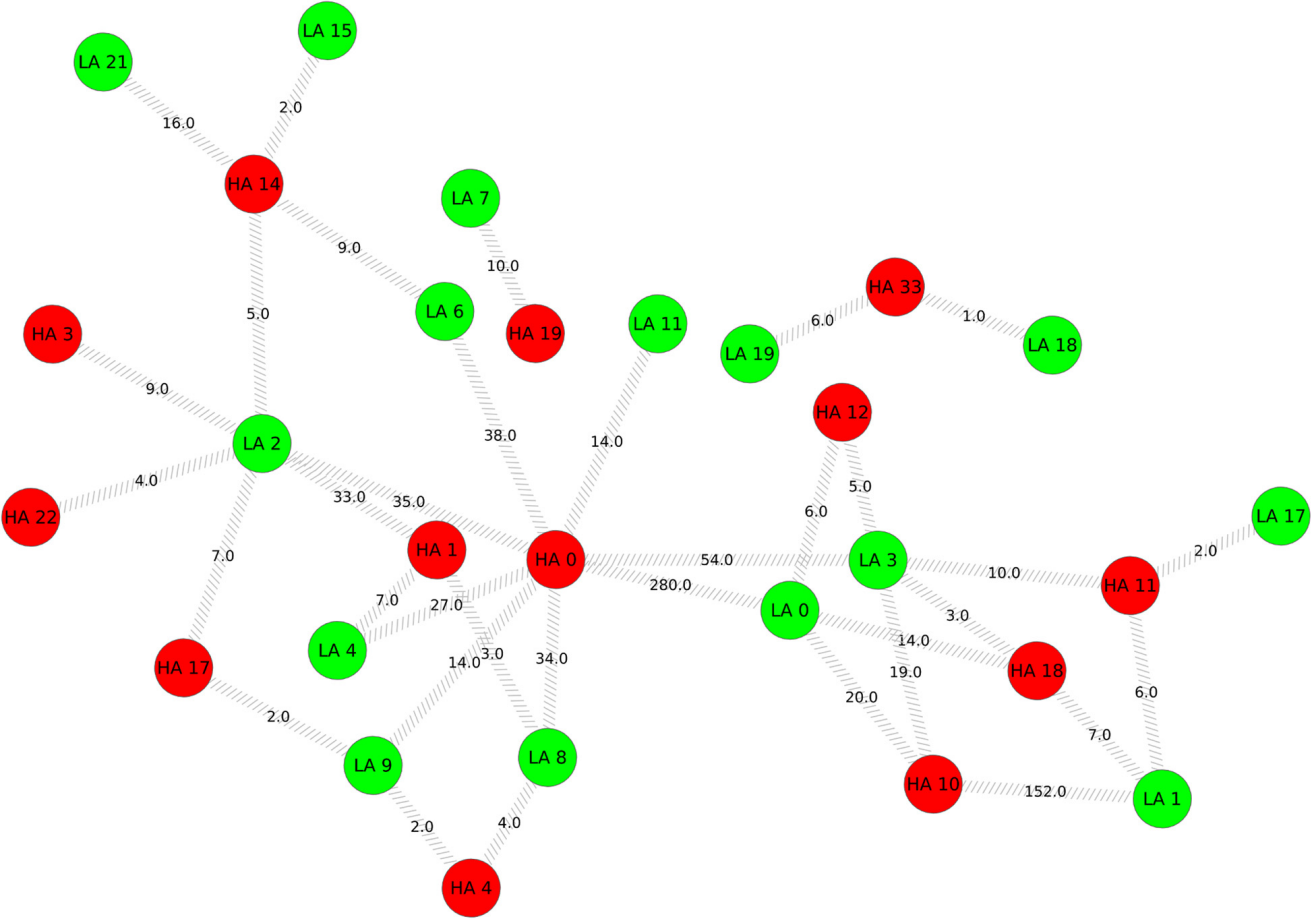
**Physical overlap between clusters.** Figure showing significant node overlap between LA and HA clusters. Legend: Green nodes indicate LA clusters and red nodes indicates HA clusters. Grey forward slashed edges indicate significant physical overlap and edge labels indicate common nodes between two clusters.

**Figure 4:**
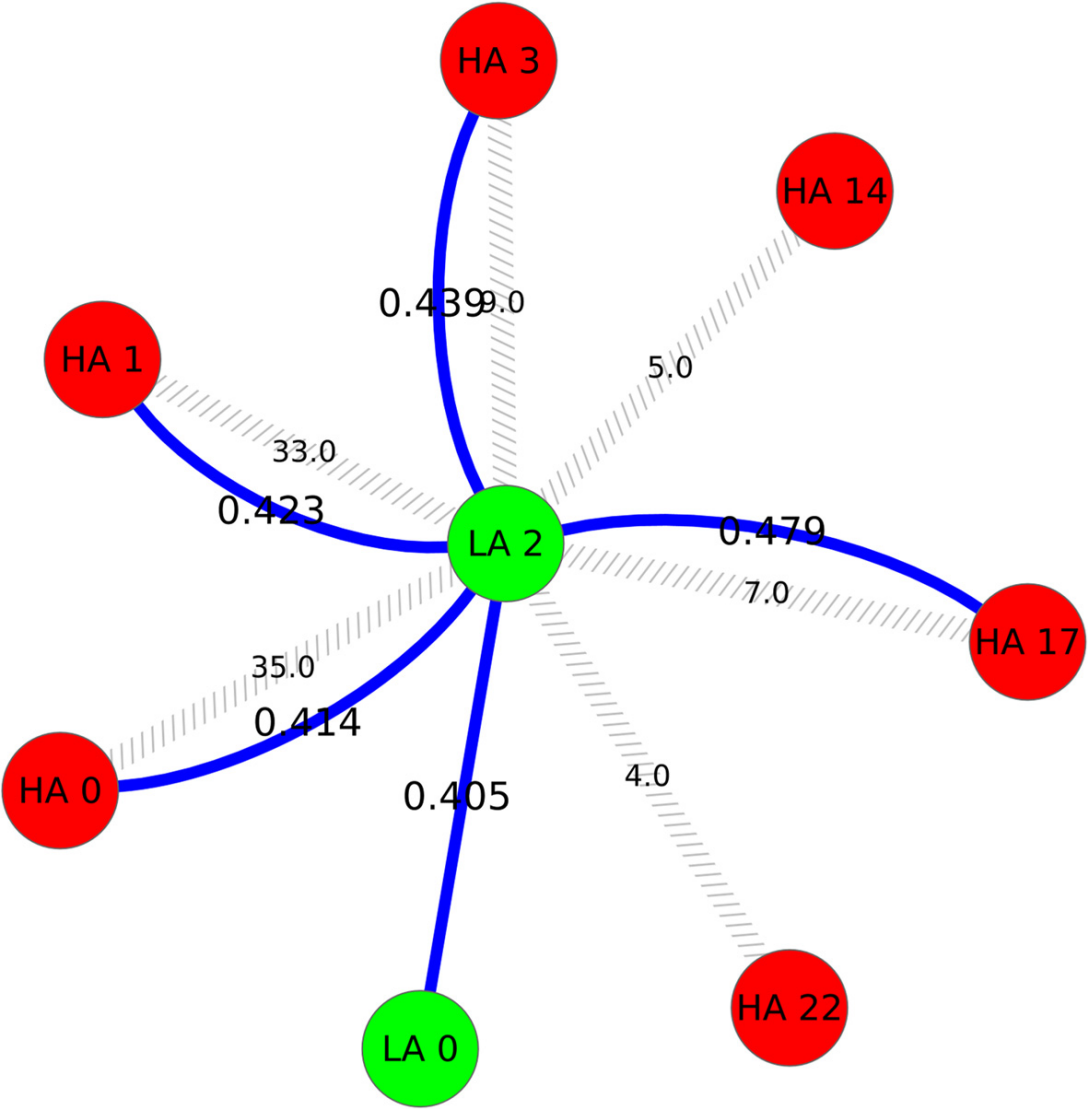
**LA 2 cluster physical and functional overlap.** Figure showing significant physical and functional overlap between LA 2 cluster and other LA and HA clusters. Legend: Green nodes indicate LA clusters and red nodes indicates HA clusters. Grey forward slashed edges indicate significant physical overlap and solid blue edges indicate functional similarity and edge labels denote the functional similarity (GO semantic similarity).

**Figure 5:**
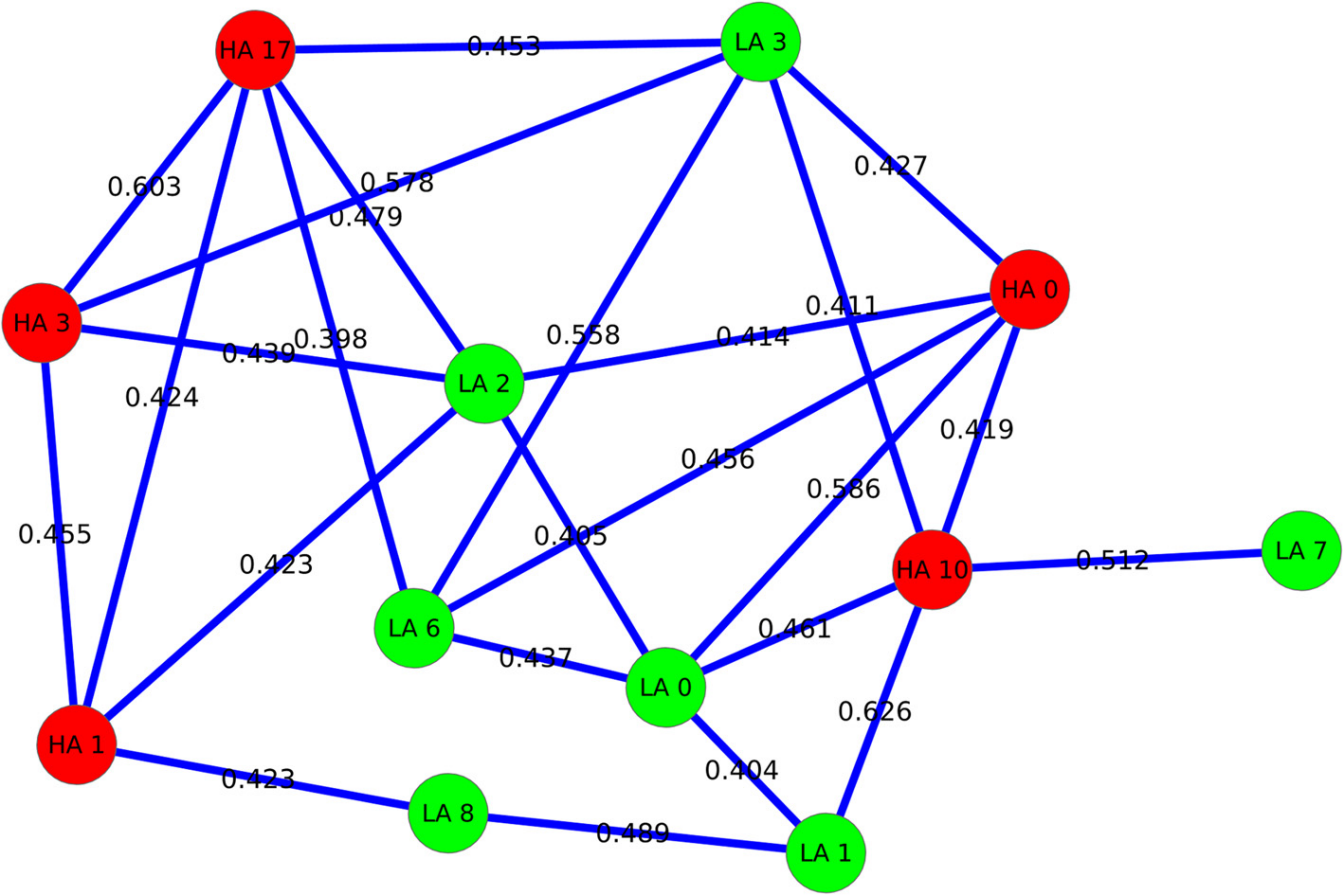
**Cluster functional overlap.** Figure showing significant functional overlap between LA and HA clusters. Legend: Green nodes indicate LA clusters and red nodes indicates HA clusters. Solid blue edges indicate functional similarity and edge labels denote the functional similarity (GO semantic similarity).

Hypergeometric test for cluster node overlap assessment showed that 15 LA clusters and 13 HA clusters had significant node overlap between them (Figure 3). The highest node overlap was between clusters LA 0 and HA 0 with 280 common nodes followed by the overlap between clusters LA 1 and HA 10 with 152 common nodes (Figure 3). LA cluster 2 showed significant node overlap between 6 HA clusters: HA 0, HA 1, HA 3, HA 14, HA 17 and HA 22. Among these clusters, the highest overlap was with cluster HA 0, with 35 nodes in common whereas HA cluster 1 with 33 common nodes showed the next highest overlap with LA cluster 2 (Figure 4). It can also be seen from Figure 4 that LA cluster 2 showed the least physical overlap with HA cluster 22 with only 4 nodes in common. The results from functional similarity assessment showed that 12 LA and HA clusters had significant functional similarity overlap (Figure 5). Out of these 12 clusters, 7 clusters were from LA network and 5 clusters were from HA network. The highest functional similarity (0.626) was between clusters LA 1 and HA 10 (Figure 5). These clusters also showed the second highest physical similarity (node overlap) (Figure 3). The second highest functional similarity (0.603) was between clusters HA 3 and HA 17, indicating that irrespective of having no physical overlap, the clusters showed significant functional similarity. The third highest functional similarity (0.586) was between clusters LA 0 and HA 0, the clusters with highest physical overlap (Figure 5, Figure 3). LA cluster 2 showed significant functional similarity with one LA cluster, LA 0 and 4 HA clusters: HA 0, HA 1, HA 3 and HA 17. Interestingly, the four HA clusters with significant functional similarity also showed significant physical similarity (node overlap) with LA cluster 2 (Figure 4).

### Sanity check

To test whether member genes of LA cluster 2 can be retrieved from microarray datasets alone, we repeated the experiment only on Duroc and Landrace (microarray) datasets and compared the resulting significant clusters to significant LA clusters. The clusters were compared using a hypergeometric test as mentioned above and the results are given in Table 6. The low androstenone microarray clusters were termed LA Duroc Landrace clusters and high androstenone clusters were termed HA Duroc Landrace clusters. The significance values for these clusters are available in Additional file 6. The results show that one microarray cluster, LA Duroc Landrace cluster 5 is highly similar to LA cluster 2. Table 6 shows that LA Duroc Landrace cluster consisted of 90 genes and out of this 87 genes were present in LA cluster 2. Since the number of genes in microarray array cluster (LA Duroc Landrace 5) was lower comparison to the number of genes in LA cluster 2, we performed GO enrichment analysis to understand the functions of this microarray cluster. Table 7 shows the results of GO enrichment analysis for LA Duroc Landrace cluster 5. The complete GO enrichment results for microarray clusters are given in Additional file 7. GO enrichment results of LA Duroc Landrace cluster 5 shows that this cluster is functionally highly similar to LA cluster 2 although smaller in size. We assume that this difference in the number of genes in LA cluster 2 and LA Duroc Landrace cluster 5 is primarily due to the effect DuF2 (RNA-seq) correlation ranks on the clustering process. This sanity check step leads to two important conclusions: (i) among the three datasets, the rank probabilities from RNA-seq dataset DuF2 has a high effect on the clustering process in comparison to the other microarray datasets and (ii) despite the smaller size of LA Duroc Landrace cluster 5, this cluster remained functionally highly similar to LA cluster 2, which shows that even after the removal of one of the datasets, the genes with high co-expression in LA cluster 2 remained as a single cluster and could be the functional core playing a major role in androstenone metabolism in low androstenone animals. Additional file 8 contains Cytoscape.xgmml network representation of the cluster LA Duroc Landrace 5 and each edge in this cluster is annotated with correlation coefficients from the microarray datasets and joint cumulative density probability calculated.

**Table 6:** **LA and microarray cluster comparison**

**Cluster 1**	**Cluster 2**	**#Cluster 1**	**#Cluster 2**	**Common genes**	**Pval**
LA 1	LA Duroc Landrace 1	316	539	287	0.0000
LA 2	LA Duroc Landrace 5	134	90	87	0.0000
LA 3	LA Duroc Landrace 1	116	539	86	0.0000
LA 4	LA Duroc Landrace 3	96	150	76	0.0000
LA 6	LA Duroc Landrace 2	86	215	67	0.0000
LA 6	LA Duroc Landrace 4	86	90	5	0.0403
LA 7	LA Duroc Landrace 6	87	70	60	0.0000
LA 7	LA Duroc Landrace 7	87	55	5	0.0059
LA 8	LA Duroc Landrace 4	72	90	60	0.0000
LA 9	LA Duroc Landrace 1	68	539	55	0.0000
LA 11	LA Duroc Landrace 2	61	215	43	0.0000
LA 11	LA Duroc Landrace 7	61	55	9	0.0000
LA 12	LA Duroc Landrace 3	40	150	32	0.0000
LA 14	LA Duroc Landrace 9	39	39	18	0.0000
LA 17	LA Duroc Landrace 3	21	150	6	0.0001
LA 17	LA Duroc Landrace 7	21	55	13	0.0000
LA 19	LA Duroc Landrace 9	20	39	17	0.0000
LA 21	LA Duroc Landrace 12	21	25	20	0.0000
LA 25	LA Duroc Landrace 13	10	11	7	0.0000
LA 1	HA Duroc Landrace 2	316	256	148	0.0000
LA 2	HA Duroc Landrace 1	134	331	47	0.0000
LA 2	HA Duroc Landrace 5	134	51	28	0.0000
LA 2	HA Duroc Landrace 8	134	27	5	0.0016
LA 2	HA Duroc Landrace 9	134	15	5	0.0001
LA 3	HA Duroc Landrace 2	116	256	29	0.0000
LA 4	HA Duroc Landrace 1	96	331	29	0.0000
LA 4	HA Duroc Landrace 3	96	96	7	0.0070
LA 4	HA Duroc Landrace 5	96	51	6	0.0011
LA 6	HA Duroc Landrace 1	86	331	41	0.0000
LA 6	HA Duroc Landrace 6	86	44	6	0.0003
LA 6	HA Duroc Landrace 9	86	15	3	0.0034
LA 8	HA Duroc Landrace 1	72	331	35	0.0000
LA 9	HA Duroc Landrace 1	68	331	23	0.0000
LA 9	HA Duroc Landrace 3	68	96	6	0.0049
LA 14	HA Duroc Landrace 7	39	32	16	0.0000
LA 18	HA Duroc Landrace 7	28	32	6	0.0000
LA 21	HA Duroc Landrace 6	21	44	13	0.0000

This table contains hypergeometric test results for LA cluster with microarray clusters.

**Table 7:** **LA duroc landrace cluster 5 GO enrichment**

**GO.ID**	**Term**	**#Enriched genes**	**Enrichment p-value**
GO:0055114	Oxidation-reduction process	27	0.00000038
GO:0051289	Protein homotetramerization	5	0.000005
GO:0006805	Xenobiotic metabolic process	6	0.000096
GO:0009058	Biosynthetic process	31	0.00485
GO:0008203	Cholesterol metabolic process	7	0.00515
GO:0048869	Cellular developmental process	8	0.0079
GO:0042493	Response to drug	7	0.0085
GO:0046395	Carboxylic acid catabolic process	6	0.02004
GO:0006979	Response to oxidative stress	7	0.02135
GO:0006810	Transport	26	0.02311
GO:0019439	Aromatic compound catabolic process	9	0.03349
GO:0009166	Nucleotide catabolic process	6	0.03466
GO:0044255	Cellular lipid metabolic process	21	0.03936
GO:0044281	Small molecule metabolic process	35	0.04629

This table contains enriched GO biological process terms for LA cluster 2 genes.

Consolidating our analysis results, we propose that the combined action of majority of the LA cluster 2 member genes might be contributing to hepatic androstenone and androgen metabolism in the LA porcine populations used in our study. Since these results are based on gene expression data from three pig populations (datasets), we further postulate that majority of the genes in this co-expression cluster might be functioning in a similar manner in all the three pig population used in our study. A drawback with the current study is that the existence of this cluster is shown only in three pig population and in addition this study was not able to provide concrete answers on hepatic androstenone metabolism in low androstenone boars. Since the comparison test show that DuF2 (RNA-seq) dataset has an effect on the clustering process, further large scale studies encompassing data from multiple porcine population and additional experiments at the genome, proteome and metabolome level are necessary to prove the validity of this cluster.

## Conclusions

Accumulation of androstenone and skatole are the major factors contributing to boar taint. The major aim of this work was to study the similarities in hepatic gene expressions in three porcine populations with similar androstenone phenotype and to identify the signature co-expression cluster(s) responsible for hepatic androstenone metabolism in these population. For this purpose, we merged metadata from three different porcine gene expression studies on three different populations using rank order statistics. The resulting networks were clustered using a state of the art clustering technique and statistically significant co-expression clusters were identified from these networks. Based on the results from enrichment analysis we hypothesize that LA cluster 2 in our results might be a signature co-expression cluster for androstenone metabolism in low androstenone animals. Our cluster similarity assessments reveal that LA cluster 2 show moderate physical and functional similarity with several HA clusters, but based on these results we further postulate that the strong co-expression and cluster behavior exhibited by LA cluster 2 member genes in low androstenone dataset might be lacking in high androstenone dataset, thus making this cluster (LA cluster 2) a prime candidate for further detailed analysis. Although the comparison test indicate that the RNA-seq correlation ranks have a large effect on the clustering process, the hypergeometric test and GO enrichment LA Duroc Landrace cluster 5 showed that this cluster was highly similar to LA cluster 2. The comparison test showed that even after removing one of the datasets from analysis, thus reducing the number of genes in the cluster, the functional enrichment remained highly similar. This shows that the co-expression of genes in this cluster is not a random effect, but the correlation ranks from DuF2 dataset has a large effect on the clustering process. This variation in the number of genes in the cluster indication of the effect of technical variabilities in high throughput results and shows the importance of validating this cluster on additional datasets from multiple pig populations.

To our knowledge, this study is one of the first attempt in porcine androstenone research community to understand population similarity in gene expression patterns based on co-expression networks. With this study, we aim to provide a baseline co-expression cluster focusing on population similarity in gene expression patterns. This cluster can further be expanded or challenged based on analysis results from other porcine populations or breeds with similar androstenone phenotypes. In order to understand the breed differences in androstenone metabolism, as a first step it is crucial to know the breed similarities in androstenone metabolism. By validating the existence of majority of the genes in this cluster in various pig breeds it would be possible to eliminate the breed specific genes from the cluster and obtain a cluster of genes common for all the pig population. Once we obtain such a common cluster, it would be possible to rank the genes in the cluster based on either their correlation coefficients/joint CDF to other genes in the cluster or based on expression values from high-throughput results. In the final step the ranking of these genes can be used as starting point for screening the animals. To conclude, we propose our co-expression cluster as one of the first attempt towards understanding gene expression similarities in hepatic androstenone metabolism. It is necessary to further validate this cluster in additional porcine populations (breeds) and to understand the potential roles of member genes in androstenone metabolism. For this purpose large scale experiments including data from multiple porcine population combining data from genomic, proteomic and metabolomic experiments are necessary.

## Additional files

Additional file 1
**Data density plots for DuF2, Duroc and Landrace datasets.** Additional file containing data density plots.

Additional file 2
**Pictorial representation of analysis workflow used.** Legend: White parallelograms with grey outline: Input/output data and results. White cylinders with red outline: data from external databases. Rectangles with light blue shades: various tools and analysis processes used in this workflow.

Additional file 3
**LA GO and KEGG enrichment table.** Additional table containing GO and KEGG enrichment analysis results for LA clusters.

Additional file 4
**HA GO and KEGG enrichment table.** Additional table containing GO and KEGG enrichment analysis results for HA clusters.

Additional file 5
**LA cluster 2 network file.** Cytoscape (.xgmml) file for LA cluster 2. Each edge in the network is annotated with correlation coefficients in all the three datasets and joint CDF calculated. This file can be visualized in Cytoscape (http://www.cytoscape.org/) following the manual for importing xgmml files into Cytoscape (http://wiki.cytoscape.org/GettingStarted).

Additional file 6
**Microarray cluster statistics.** Additional table containing statistics for microarray clusters.

Additional file 7
**Microarray cluster GO enrichment.** Additional table containing GO enrichment results for microarray clusters.

Additional file 8
**LA Duroc Landrace cluster 5 network file.** Cytoscape (.xgmml) file for LA Duroc Landrace cluster 5. Each edge in the network is annotated with correlation coefficients in the microarray datasets and joint CDF calculated. This file can be visualized in Cytoscape (http://www.cytoscape.org/) following the manual for importing xgmml files into Cytoscape (http://wiki.cytoscape.org/GettingStarted).
