# Supplementary material for: Identification of gene co-expression clusters in liver tissues from multiple porcine populations with high and low backfat androstenone phenotype
Source: BMC Genet. 2015 Feb 28;16:21. doi: 10.1186/s12863-014-0158-8 (PMC4365963; doi:10.1186/s12863-014-0158-8)
Supplement: Additional file 1 — Data density plots for DuF2, Duroc and Landrace datasets. Additional file containing data density plots. [file 12863_2014_158_MOESM1_ESM.pdf]

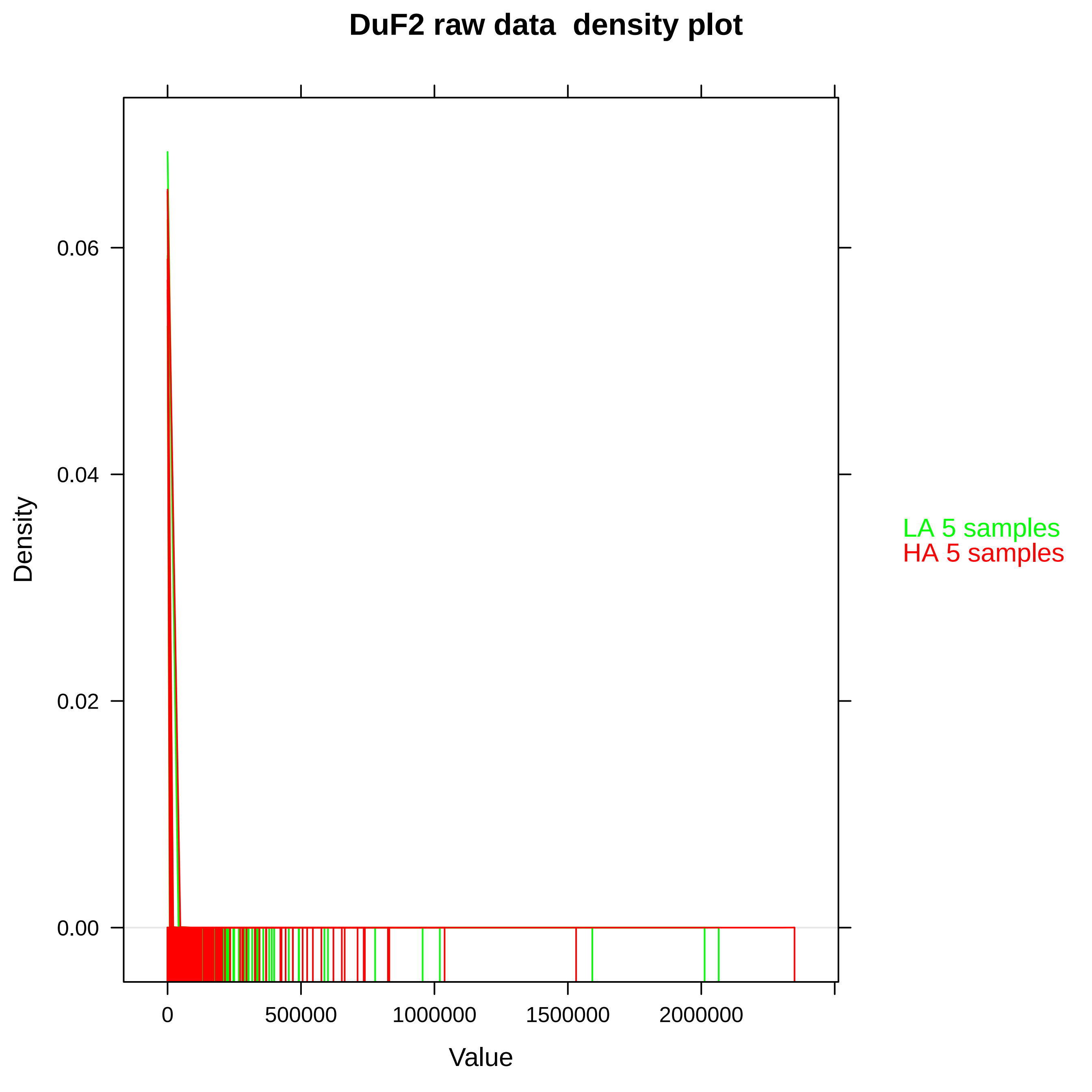
DuF2 raw data density plot
Density
0.06
0.04
0.02
0.00
0
500000
1000000
1500000
2000000
Value
LA 5 samples
HA 5 samples

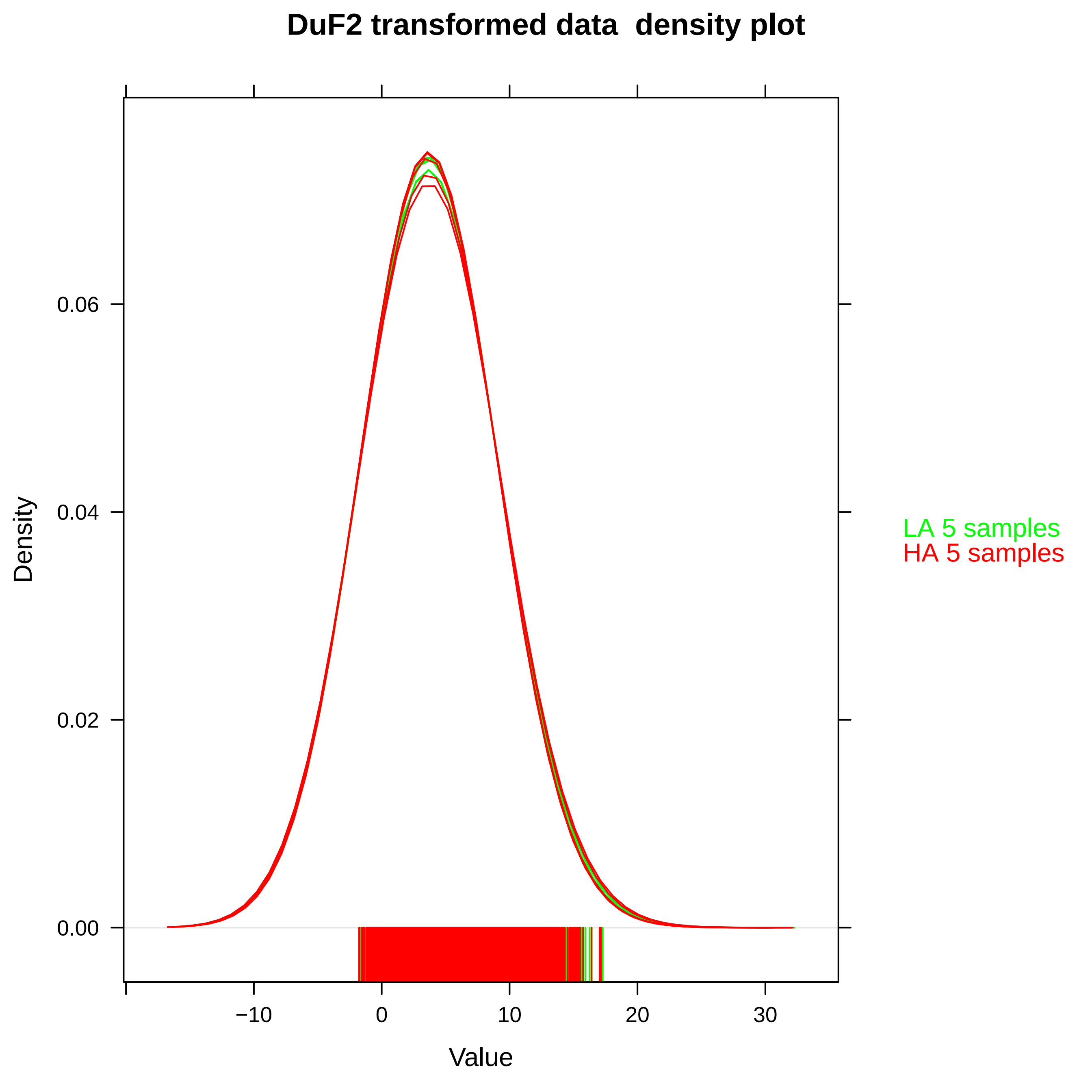
DuF2 transformed data density plot
0.06
0.04
0.02
0.00
Density
−10
0
10
20
30
Value
LA 5 samples
HA 5 samples

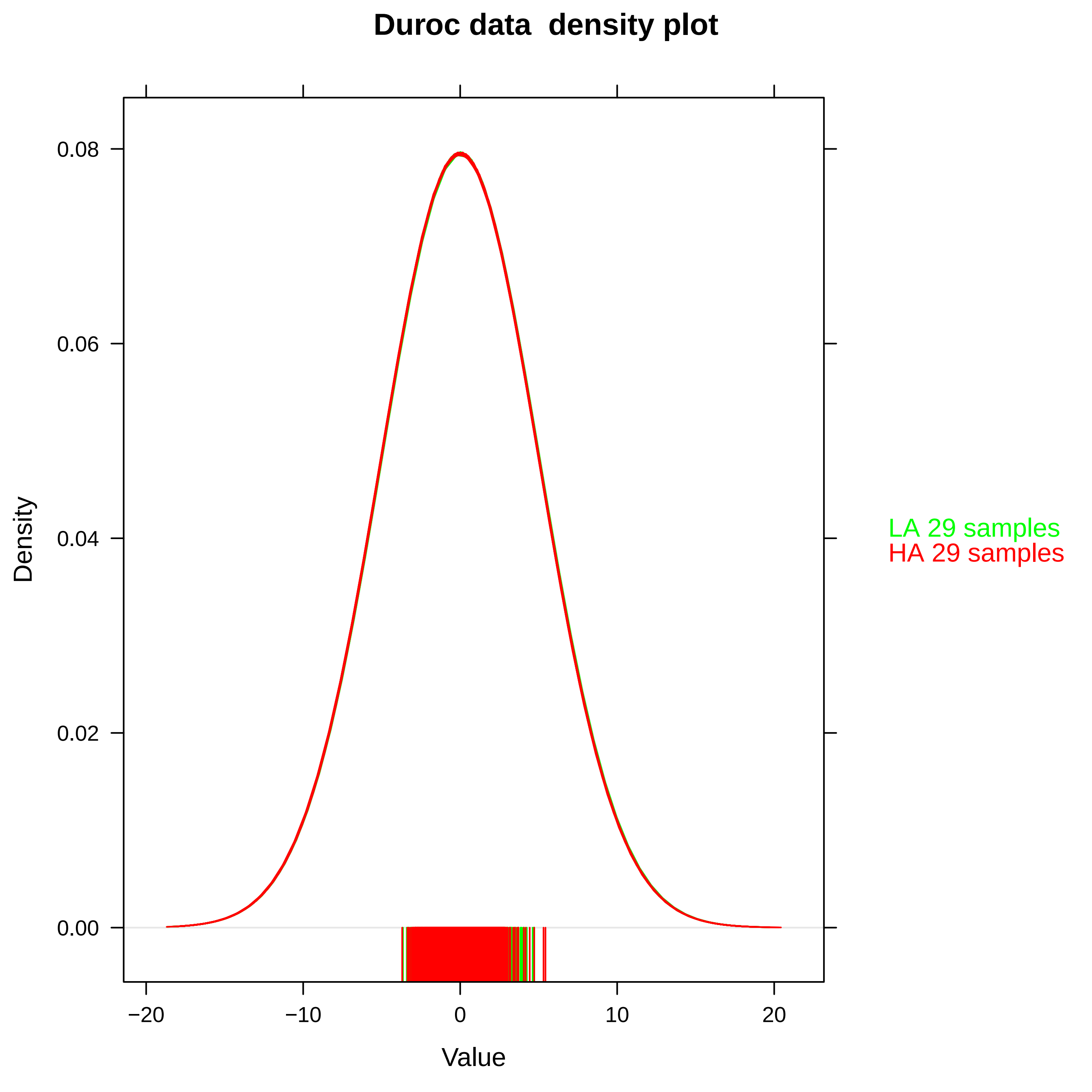
Duroc data density plot
Density
0.00
0.02
0.04
0.06
0.08
−20
−10
0
10
20
Value
LA 29 samples
HA 29 samples

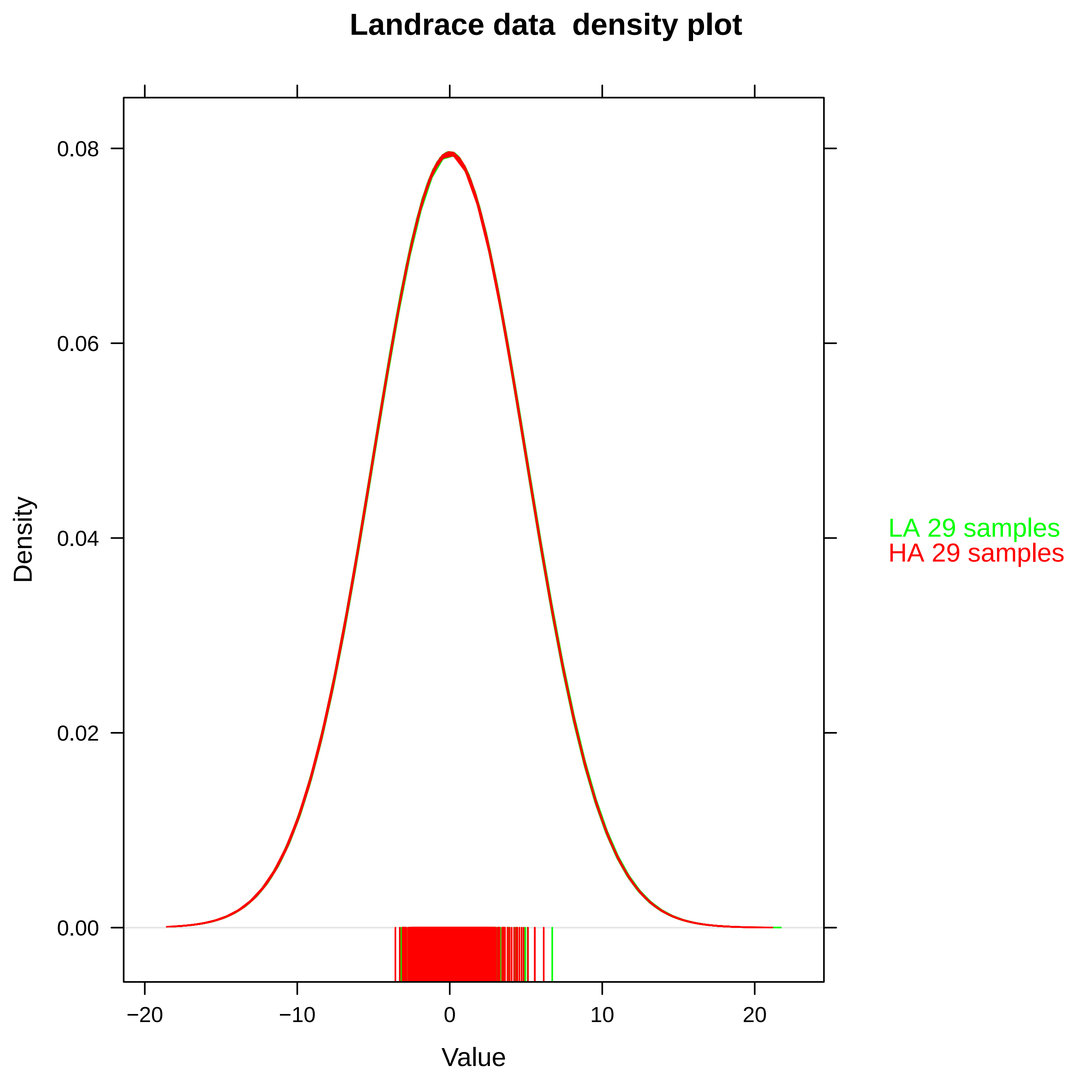
Landrace data density plot
Density
0.08
0.06
0.04
0.02
0.00
−20
−10
0
10
20
Value
LA 29 samples
HA 29 samples
