# Supplementary material for: Identification of gene co-expression clusters in liver tissues from multiple porcine populations with high and low backfat androstenone phenotype
Source: BMC Genet. 2015 Feb 28;16:21. doi: 10.1186/s12863-014-0158-8 (PMC4365963; doi:10.1186/s12863-014-0158-8)
Supplement: Additional file 2 — Pictorial representation of analysis workflow used. Legend: White parallelograms with grey outline: Input/output data and results. White cylinders with red outline: data from external databases. Rectangles with light blue shades: various tools and analysis processes used in this workflow. [file 12863_2014_158_MOESM2_ESM.pdf]

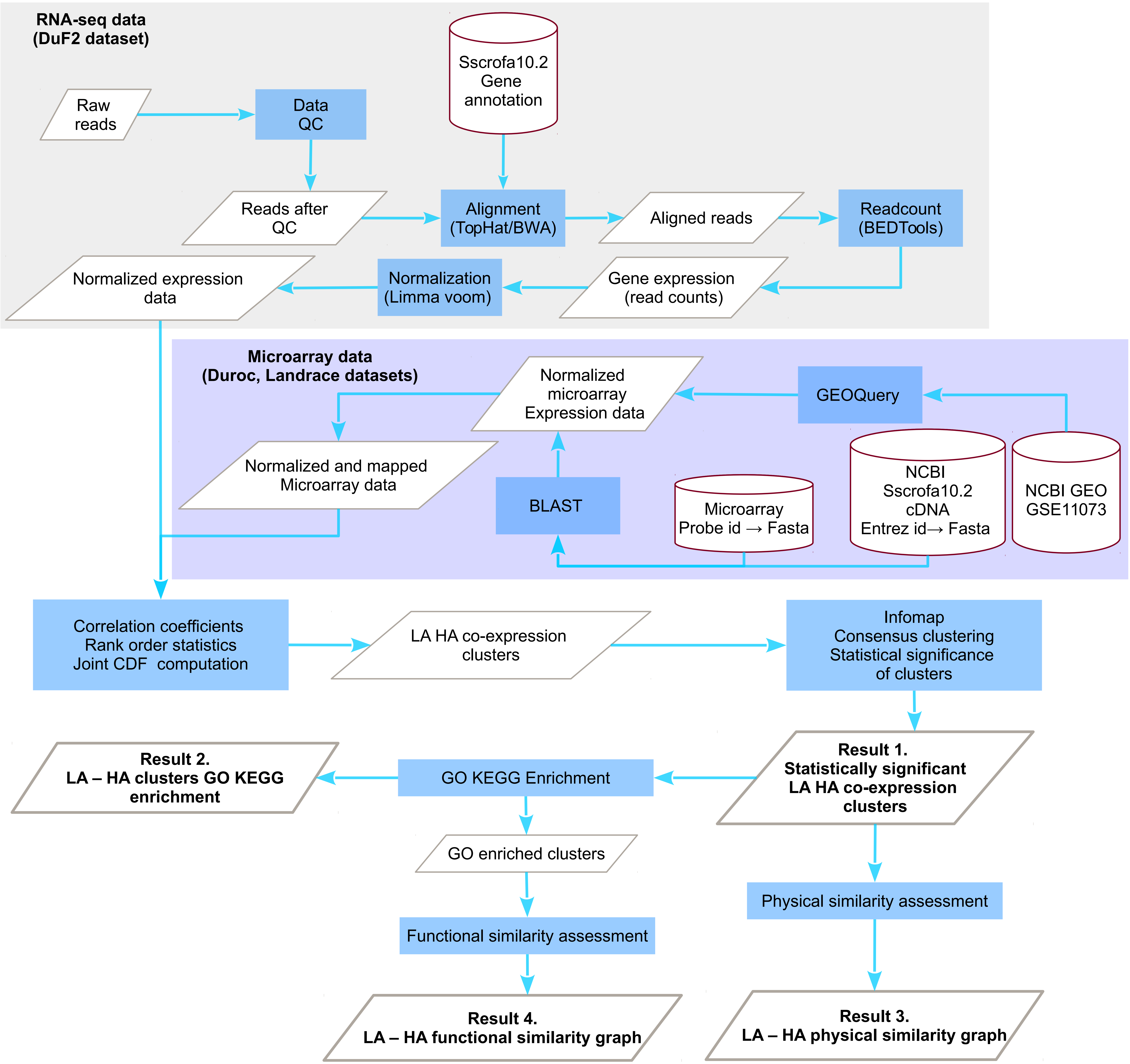
RNA-seq data
(DuF2 dataset)
Raw reads
Data QC
Sscrofa10.2 Gene annotation
Reads after QC
Alignment (TopHat/BWA)
Aligned reads
Readcount (BEDTools)
Normalized expression data
Normalization (Limma voom)
Gene expression (read counts)
Microarray data
(Duroc, Landrace datasets)
Normalized microarray Expression data
GEOQuery
Normalized and mapped Microarray data
BLAST
Microarray Probe id → Fasta
NCBI Sscrofa10.2 cDNA Entrez id→ Fasta
NCBI GEO GSE11073
Correlation coefficients Rank order statistics Joint CDF computation
LA HA co-expression clusters
Infomap Consensus clustering Statistical significance of clusters
Result 2. LA – HA clusters GO KEGG enrichment
GO KEGG Enrichment
Result 1. Statistically significant LA HA co-expression clusters
GO enriched clusters
Physical similarity assessment
Functional similarity assessment
Result 4. LA – HA functional similarity graph
Result 3. LA – HA physical similarity graph
